# A population-based study of chronic neutrophilic leukemia in the United States

**DOI:** 10.1038/s41408-020-0334-1

**Published:** 2020-06-15

**Authors:** Gordon J. Ruan, Caleb J. Smith, Courtney Day, William S. Harmsen, Darci L. Zblewski, Hassan Alkhateeb, Kebede Begna, Aref Al-Kali, Mark R. Litzow, William Hogan, Natasha Szuber, Naseema Gangat, Mrinal S. Patnaik, Animesh Pardanani, Michelle A. Elliott, Ayalew Tefferi, Ronald S. Go, Mithun V. Shah

**Affiliations:** 1 Division of Hematology, Mayo Clinic, Rochester, MN USA; 2 Division of Biomedical Statistics and Informatics, Mayo Clinic, Rochester, MN USA

**Keywords:** Epidemiology, Myeloproliferative disease

Dear Editor,

Chronic neutrophilic leukemia (CNL) is a rare and aggressive *BCR-ABL1* (Philadelphia chromosome) negative myeloproliferative neoplasm that carries a poor prognosis. It is defined by persistent mature neutrophilic leukocytosis, bone marrow granulocytic hyperplasia, and hepatosplenomegaly^1^. Despite the 2013 seminal discovery of the oncogenic driver mutations in colony-stimulating factor 3 receptor (*CSF3R*) in CNL^2^, no standard of care exists. Chemotherapy is not able to provide durable responses, the literature on allogeneic stem cell transplantation (ASCT) is scarce, and targeted therapies such as JAK-2 inhibitors and dasatinib are still being studied for efficacy and safety^1,3,4^. There is a paucity of population-based data on CNL and contemporary clinical data are limited to institutional case series. In this study, we combined the Surveillance, Epidemiology, and End Results (SEER) program and the National Cancer Database (NCDB) to study population-based outcomes for patients with CNL.

SEER is a program of the National Cancer Institute that collects and publishes cancer incidence and survival data covering ~28% of the US population^5^. The NCDB is a joint project of the Commission on Cancer of the American College of Surgeons and the American Cancer Society that is a nationwide oncology outcomes database for >1500 cancer programs in the USA and Puerto Rico, capturing 70% of all newly diagnosed cases of cancer in the USA^6^. The NCDB Participant User File and SEER 18 registries were used to identify patients with ICD-O-3 diagnosis code 9963/3 from 2004 to 2015.

Using SEER, data on incidence, overall survival (OS), relative survival, subsequent malignancies, and main causes of death were obtained. Incidence was age-adjusted to the US 2000 standard population. Relative survival was defined as the ratio of the proportion of observed survivors in a cohort of CNL patients to the proportion of expected survivors in a comparable set of individuals that did not have CNL, adjusting for the general survival of the US population for race, sex, age, and time when the diagnosis was established. Incidence rates and relative survival were calculated using the SEER*Stat software (version 8.3.6; NCI, Bethesda, MD, USA), while OS was calculated by extracting and analyzing SEER data in JMP 14 (SAS institute Inc., Cary, NC, USA).

For NCDB data, comorbid disease burden was calculated using the Deyo adaptation (1992) of Charlson’s comorbidity index, which are mapped from as many reported ICD-9-CM or ICD-10 secondary diagnosis codes^7^. Per the NCDB’s methodology, prednisone was classified as hormone therapy, interferon-alpha as immunotherapy, and hydroxyurea, cladribine, ruxolitinib, imatinib, and hypomethylating agents as chemotherapy. Treatment that could not be defined by the NCDB was deemed “other treatment”. OS was analyzed using the Kaplan–Meier method. Hazard ratios (HR) with confidence intervals (CI) were calculated using Cox proportional hazards model. Variables significant in univariate analysis were included in a multivariate analysis. Statistical analyses for NCDB were performed using SAS version 9.0.

**Fig. 1:**
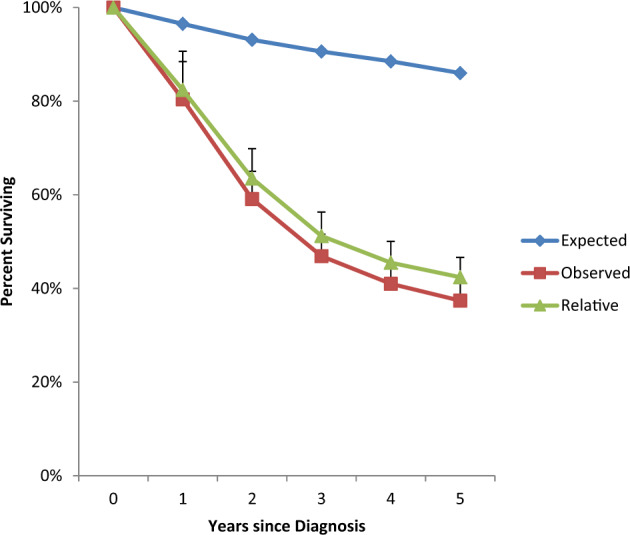
Survival curves from the SEER cohort. Expected, observed, and CNL-specific relative survival (mean ± standard error of mean).

A total of 73 patients were identified in SEER. The median age at diagnosis was 73 years (range 21–100); 44 (60%) were males. Fifty-four (74%) were non-Hispanic White, 9 were non-Hispanic Black, 6 were Hispanic, and 4 were non-Hispanic American Indian/Alaskan Native or non-Hispanic Asian Pacific Islander. The overall incidence was 0.1 cases/1,000,000 individuals. Incidence was not impacted by race or sex. The incidence of CNL did not significantly change over the years from 2004 to 2015. Twenty-six patients (36%) had prior malignancies of which three were noted to have a prior hematologic malignancy: acute myeloid leukemia (AML)^1^, myeloma^1^, and chronic myelogenous leukemia^1^. Twenty-six patients (36%) had subsequent malignancies with five hematologic malignancies: AML^4^ and nodal non-Hodgkin’s lymphoma^1^. Among the patients who had subsequent AML, the average age was 67 (range 52–80), 75% were male, and the average time to developing AML was 13 months (range 10–16). Vital status was known for all patients and there were 49 deaths (67%) at the last follow up with a median follow up of 4.3 years (interquartile range (IQR) 3.6–5.1), the median OS was 1.8 years (95% CI: 1.3–2.5). Twenty-one patients (29%) died from CNL, 19 (26%) had an unknown cause of death, and 9 (12%) died from other causes: accidents and adverse effects^2^, cerebrovascular disease^2^, chronic obstructive pulmonary disease^2^, cardiovascular disease^2^, and Alzheimer’s disease^1^. When comparing CNL patients to the US general population, 51 patients were matched to the expected survival tables. In the general US population, the expected survival for 12, 24, 36, 48, and 60 months was 97, 93, 91, 89, and 86%. In contrast, survival for patients with CNL at the same time points was 82, 64, 51, 46, and 42% (Fig. 1).

**Fig. 2:**
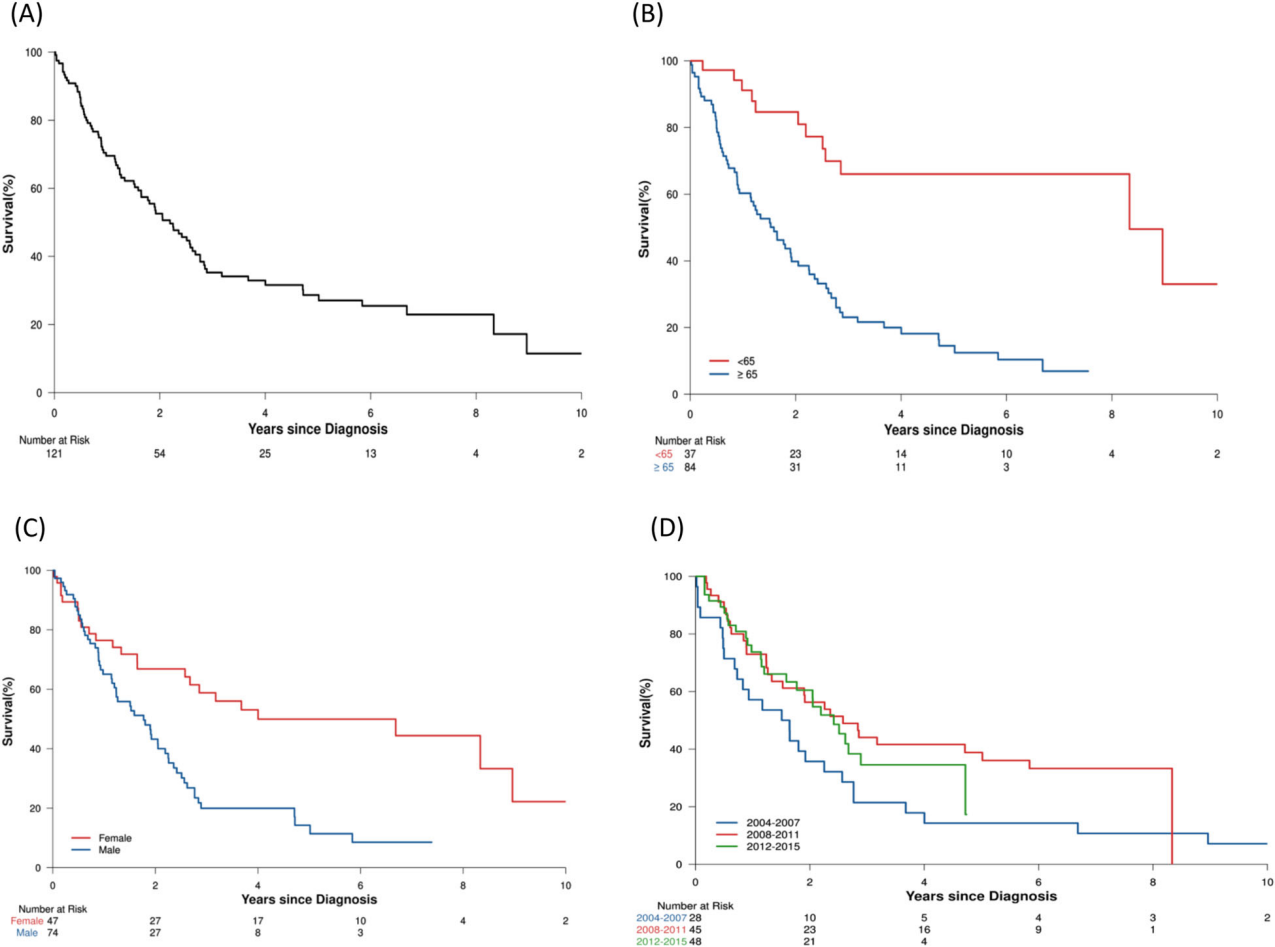
Kaplan-Meier survival estimates using the NCDB cohort. **a** Overall survival (OS) in the entire cohort. **b** OS in patients diagnosed either age ≥65 years (blue) or younger (red) at the time of diagnosis is a poor risk factor for patients with CNL. **c** Male sex (blue) is also a poor risk factor compared with females (red). **d** OS trends by the time period of diagnosis.

A total of 121 CNL patients were found in NCDB. The median age at diagnosis was 70 years (range 21–90); 74 (61%) were males and 84 (69%) were ≥65 years. The median follow up time was 5.8 years (IQR 2.9–7.4). Eighty-nine patients (73%) had a Charlson Deyo score (CDS) of 0, 19 patients (16%) with CDS of 1, and 13 patients (11%) with CDS ≥2. Eighty patients (70%) had government insurance, while 34 (30%) had private insurance. Median OS was 2.2 years (IQR 0.8–6.7) (Fig. 2a). OS was 70% at 1 year, 29% at 5 years, and 11% at 10 years. The year of diagnosis was not associated with improved OS (HR: 0.95 (95% CI: 0.88–1.02), *p* = 0.14) (Fig. 2d). Seventy-one patients (59%) received chemotherapy, 39 patients (32%) received no initial treatment, 3% received immunotherapy, 3% received “other treatment”, and 2% received hormone therapy. The median time to chemotherapy from the day of diagnosis was 15 days (IQR 7–35). The OS of those who received chemotherapy was 71% at 1- and 27% at 5-years compared with 69% and 32% at 1- and 5- years, respectively, for those who did not receive chemotherapy (*p* = 0.38). 2% received ASCT (age <65 years with CDS of 0) as the initial therapy and all were alive at 5 years. Factors predicting inferior OS on univariate analysis were age ≥65 years at diagnosis (*p* = 0.004), male (*p* = 0.006), CDS >1 (*p* = 0.04), and government insurance (*p* = 0.004). Table 1 shows the multivariate analysis and Fig. 2 shows the survival estimates.

**Table. 1:** NCDB cohort: univariate and multivariate Cox proportional hazards model.

Variables	*N* (%)	Univariate analysis			Multivariate analysis		
		HR	95% CI	*p* value	HR	95% CI	*p* value
Age ≥65 years	84 (69)	4.8	2.5–9.4	<0.001	**3.2**	**1.4**–**7.1**	**0.004**
Male	74 (61)	2.5	1.5–4.2	<0.001	**2.1**	**1.2**–**3.7**	**0.006**
CDS >1	32 (26)	1.7	1.03–2.7	0.04	1.3	0.8–2.1	0.32
Not Hispanic	106 (96)	1.4	0.4–4.3	0.6			
Non-White	12 (10)	0.5	0.2–1.2	0.1			
Government insurance	80 (70)	2.2	1.3–3.8	0.004	1.3	0.7–2.5	0.34
Chemotherapy	71 (59)	1.2	0.8–1.9	0.4			

Values in bold are statistically significant on univariate analysis AND multivariate analysis.

This is the first and the largest retrospective study on CNL and is the only study that provides data on OS trends over the years. Our study shows that CNL continues to be an incredibly rare disease. Despite the discovery of the *CSF3R* mutation in 2013, there has not been an increase in incidence. CNL has been described as a diagnosis of the elderly, and our study confirms this. However, our study also shows that a minority of patients are diagnosed before the age of 65.

The cause of death for the majority of patients was the disease as opposed to other causes. In our study, patients with CNL had a short median OS of 1.8 years in SEER and 2.2 years in NCDB, which is consistent with the median OS seen in the largest institutional case series^3^. Approximately one-third of our patient population had a prior history of malignancy, while another third of our study population developed a subsequent malignancy. The most common subsequent malignancy noted was AML (5.4%), most likely representing transformation to an aggressive phenotype. One review suggested a high number of CNL cases associated with plasma cell neoplasms^1^, but we did not see this in our population-based study.

There is currently no standard of care for CNL. In the largest institutional case series on CNL done at our institution, the majority of patients had hydroxyurea as first line^3^. Other agents that have been used as first-line therapy include interferon-alpha and splenectomy^1^. ASCT has been shown to induce remission, but this is limited to case reports and case series^4^, and ASCT is rarely used as first-line therapy. In our study, it is interesting to note that the patients who did receive ASCT as front-line therapy are still alive at 5 years, albeit they were <65 years of age and had CDS of 0.

Limitations of our studies include the inability to access individual granular data such as laboratory parameters including molecular data, and subsequent lines of treatment. However, it should be noted that our study spanned from 2004 to 2015, and activating *CSF3R* mutations were discovered in 2013 and incorporated as a major diagnostic criterion in the 2016 World Health Organization classification^2,8^. Finally, NCDB only provides info on “initial” treatment. Thus, if a patient was coded to have received no treatment, this could mean that the patient was observed because no treatment was ever required, the patient did not receive treatment even though it was recommended or necessary, or the patient received treatment as a subsequent line of therapy but the initial management was observation.

In summary, our study confirms that CNL is a rare leukemia with no curative therapies available. Despite significant research, CNL remains an incurable disease inflicting significant mortality with no improvement in outcomes over the years. Age ≥65 years at the time of diagnosis and male sex were independent predictors of inferior OS. Additional research is needed to better understand the poor prognosis of this disease and develop improved therapeutics.
